# Use of an inertial measurement unit sensor in pedicle screw placement improves trajectory accuracy

**DOI:** 10.1371/journal.pone.0242512

**Published:** 2020-11-16

**Authors:** Satoshi Baba, Kenichi Kawaguchi, Kazuhito Itamoto, Takeshi Watanabe, Mitsumasa Hayashida, Takao Mae, Yasuharu Nakashima, Go Kato

**Affiliations:** 1 Department of Spine Surgery, Saga Medical Center, Koseikan, Saga, Japan; 2 Trauma Center, Saga Medical Center, Koseikan, Saga, Japan; 3 Department of Orthopedic Surgery, Kyushu University Graduate School of Medical Sciences, Fukuoka, Japan; 4 Department of Small Animal Clinical Science, Joint Faculty of Veterinary Medicine, Yamaguchi University, Yamaguchi, Japan; 5 Department of Orthopedic Surgery, Watanabe Orthopedic Hospital, Itoshima, Fukuoka, Japan; 6 Department of Orthopedic Surgery, Saga Medical Center, Koseikan, Saga, Japan; 7 Department of Orthopedic Surgery, Fukuoka Red Cross Hospital, Fukuoka, Japan; University of California San Francisco, UNITED STATES

## Abstract

Ascertaining the accuracy of the pedicle screw (PS) trajectories is important as PS malpositioning can cause critical complications. We aimed to determine the angle range over which estimation is unreliable; build a low-cost PS placement support system that uses an inertial measurement unit (IMU) to enable the monitoring of surgical tools and PS trajectories, and determine the situations where IMU support would be most beneficial. In PS insertion experiments, we used cadaver samples that included lumbar porcine spines. Computed tomography images obtained before and after PS insertion were viewed. Offsets between the planned and implanted PS trajectories in the freehand and IMU-assisted groups were analyzed. The PS cortical bone breaches were classified according to the Gertzbein and Robbins criteria (GRC). Added head-down tilted sample experiments were repeated wherein we expected a decreased rostro-caudal rotational accuracy of the PS according to the angle estimation ability results. Evaluation of the PS trajectory accuracy revealed no significant advantage of IMU-assisted rostro-caudal rotational accuracy versus freehand accuracy. According to the GRC, IMU assistance significantly increased the rate of clinically acceptable PS positions (RoCA) than the freehand technique. In the head-down tilted sample experiments, IMU assist provided increased accuracies with both rostro-caudal and medial rotational techniques when compared with the freehand technique. In the freehand group, RoCA was significantly decreased in samples with rostral tilting relative to that in the samples without. However, In the IMU-assisted group, no significant difference in RoCA between the samples with and without head-down tilting was observed. Even when the planned PS medial and/or rostro-caudal rotational angle was relatively large and difficult to reproduce manually, IMU-support helped maintain the PS trajectory accuracy and positioning safety. IMU assist in PS placement was more beneficial, especially for larger rostro-caudal and/or medial rotational pedicle angles.

## Introduction

Since lumbar spinal immobilization using pedicle screws (PSs) was conceived by Boucher [1], the indications for PS placement have been broadly expanded to include cervical spinal fixation [2]. PS mispositioning, however, can cause critical acute complications, such as injury to the spinal cord, nerve root, and vasculature or cause cerebrospinal fluid leaks [3–5] in addition to mechanical fixation failures requiring revision surgery [6–8]. The number of spinal fusion surgeries using PS placement is expected to increase significantly, especially in countries with aging societies.

For safe and accurate PS placement, both the coordinates of the entry point (EP) and PS trajectory are critical, and many studies have used 3-dimensional CT-based navigation system to demonstrate improved accuracy for these parameters [9,10]. There has been significant evolution in spinal surgery navigation systems over the past decade. For example, other newly developed PS placement support tools, such as spine-mounted robot-assisted systems [11–15] or artificial reality spine surgical navigation system [16–18] have been introduced.

Although a large variation in PS placement accuracy was demonstrated in previous papers, a meta-analysis with a large sample size found that the median rates of PS malposition were 4.8% with use of contemporary benchmark CT-based navigation and 9.7% without navigation [19]. The analysis concluded that navigation systems provided highly accurate PS placement except at the thoracic levels.

Despite the benefits of navigation systems, adoption of navigation as a standard of care has been slow because of their cost, setup and registration times, and interruption of surgical workflow [20–22]. On the other hand, C-arms are simple to use and widely available, but they usually allow monitoring of one plane and staff are exposed to radiation [23].

Of the parameters related to reproducibility of preoperatively planned PS placement, the EPs, especially of the thoracic and lumbosacral spine, can be identified easily by measuring the distances from anatomical landmarks [24]. However, the PS trajectory could not be monitored easily without any device support. The study aim was to increase the reproducibility of preoperatively planned PS placement in porcine lumbar spines by using a low-cost micro-electromechanical system (MEMS)-based inertial measurement unit (IMU) to perform real-time monitoring of the trajectories of surgical tools and PSs. IMUs comprise an acceleration sensor and gyroscope and are used to detect the acceleration and angular velocity of objects. In addition to their common use in motion-sensitive applications for tablets and smartphones, IMUs are used to enhance the accuracy of total knee arthroplasty [25]. Additionally, a technology that combines IMUs and fluoroscopy for percutaneous placement of lumbar and sacral PSs also has been developed [26].

## Materials and methods

The study protocol was reviewed and approved by the Research Ethics Committee of the Saga Medical Center Koseikan. In the PS insertion experiments, 23 *ex vivo* specific-pathogen-free specimens, including L1–6 lumbar spines and paraspinal muscles harvested from 10-week-old female pig cadavers, were obtained from Intervention Technical Center (Kobe, Japan).

### Evaluation of the angle estimation ability

Initially, we evaluated the human ability to estimate an angle. A lack of accurate angle estimation ability could be a problem in reproducing the preoperatively planned trajectories of surgical tools in PS placement. Two of the authors (S.B. and G.K.) along with 10 naïve observers (7 men and 3 women) participated in the experiment. Because we assumed that angle estimation ability was not developed directly due to spine surgery and was not related to the years of experience in spine surgery, we corrected the data using the findings of 10 volunteers who were not spine surgeons, including four orthopedic surgeons, two residents, one nurse and three nonmedical personnel. The detailed evaluation method has been described in the supporting information (S1 File).

### Custom-made IMU-mounted jig

We made a wireless IMU (TAG250N2020; approximately 1000 USD; Tamagawa Seiki, Iida, Nagano, Japan) mounted jig for monitoring the 3-dimensional orientation of a pedicle probe and screwdriver. The jig consisted of a flat platform for fixing the IMU, two handles that were parallel to two axes of the IMU for adjusting rostro-caudal and medio-lateral angulations, and an outer cylinder that was perpendicular to the platform (S2 Fig).

The diameters of the inner cylinders of the IMU-mounted jigs were first designed to match those of the pedicle probe and screwdriver, and the inner part of the cylinders was filed to permit these surgical instruments to be forwarded smoothly. The wiggling room between the IMU-mounted jig cylinder and surgical instruments was almost negligible, and the trajectories of those instruments were considered identical with those measured using the IMU.

The jig was made with low-cost PA12GB material by using a 3D printer (Multi Jet Fusion, Hewlett Packard, Palo Alto, CA, USA). Because the aim of this study was to preliminarily assess whether IMU assist could increase the accuracy of a PS position in an *ex vivo* experiment, we were not concerned about waterproofing or heat-resistant properties necessary for sterilization. The trajectory of the IMU fixed on the jig was shown on the monitor of a wirelessly connected PC. Because vertebral rotation could occur during pedicle probing and screwing, the orientation was checked when probing or screwing was not performed.

#### PS insertion in a porcine lumbar spine

CT scans using a 64-row multi-detector CT unit with a slice thickness of 0.6 mm of the lumbar spine before and after PS insertion surgery were acquired and viewed in the multiplanar reconstruction mode (MPR) on a ZioCube DICOM viewer (Ziosoft Co. Ltd., Japan).

Extracted PSs from patients were reused (4.5–7.5 mm, 0.5-mm increments). We selected the largest but ≥1 mm smaller size screws from our screw stocks. For example, if the pedicle diameter was 7.3 mm, we selected a 6.0-mm screw, and if the pedicle diameter was 8.0 mm, we selected a 7.0-mm screw. Because we focused on the screw placement accuracy at the pedicle, the screw length was not considered.

### Preoperative planning

All screw trajectories were planned by a senior spine surgeon. In this study, three axes and the motion about each were defined as follows (S3 Fig):

1. Perpendicular axis
  The perpendicular axis was perpendicular to the ground, parallel to the outer cylinder of the jig for the pedicle probe and screwdriver. The motion about this axis was kept to zero while probing or screwdriving.
2. Transverse axis
  The transverse axis was perpendicular to the sagittal axis of the spinous process tip and the perpendicular axis, parallel to the one jig handle. Motion about this axis creates rostral or caudal tilting.
3. Sagittal axis
  The sagittal axis was perpendicular to the transverse axis and perpendicular axis parallel to the one jig handle and sagittal axis of the spinous process tip. The motion about this axis creates medial or lateral tilting.

Initially, the ideal trajectory of PS placement in this study was defined as a line parallel to the axis of the narrowest portion of the pedicle on an arbitrary axial plane (AAP) and parallel to the cranial endplate of the vertebral body (CE), which was then bisected. The EP was defined as the point at which the ideal trajectory and rostral articular process base crossed.

The angle between the AAP parallel to the CE on which the ideal trajectory was defined (plane filled with gray color) and the wood board surface on which the cadaver was mounted (α in S3 Fig) was measured on a DICOM viewer. Additionally, the angle of medial tilting of the ideal trajectory on the AAP parallel to the CE (β in S3 Fig) was measured. The angle, γ, between the ideal trajectory and board surface was calculated according to the following formula:

tanβ=tanγ/(1/cosα)

γ(degrees,°)=atan(tanβ/cosα)=360/2πxatan(tan(2πβ/360)/cos(2πα/360))

When performing IMU-assisted PS placement, a calibration was performed after the outer cylinder was held perpendicular to the ground and one of the two handles was held macroscopically parallel to the posterior spinous line (i.e., tips of the spinous process). We aimed to reproduce these α and γ angles demonstrated by the IMU during pedicle probing and screwing. When observing the CT scan of the porcine lumbar spines, a scoliotic curve or osteoarthritic change was not observed in all cases; therefore, the plane on which the upper vertebral endplates were included and the plane on which the posterior spinous lines were included were almost orthogonal. Therefore, after the calibration, the 3 IMU axes were supposed to be a line perpendicular to the ground, a line perpendicular to line 1 on a plane on which the posterior spinous processes existed, and a line perpendicular to lines 1 and 2 (which was almost parallel to the vertebral body endplate). In this case, the plane on which lines 1 and 2 existed was the sagittal plane, and the plane on which lines 1 and 3 existed was the axial plane.

To increase the accuracy of the EP location, EP coordinates, the lateral tip of the transverse process (TTP) lateral tip, and rostral tip of the rostral articular process (TRAP) were measured by using a DICOM viewer. The distances from the TTP to EP and TRAP to EP were calculated. A compass was used to find an intersection of arcs with the radii of those distances (S4 Fig).

### Procedure for PS placement by freehand and the IMU-assisted technique

In one porcine lumbar spine sample, all PSs on one side were placed by one of the authors (S.B., 1 year of spine surgery experience). On the contralateral side, different author (G.K., 6 years of spine surgery experience) continuously checked the 3-dimensional direction of the outer jig cylinder shown on the PC monitor while the pedicle probe tip inserted into the outer cylinder was pointed at the EP described above. Another author (S.B.) pushed a pedicle probe forward through the outer cylinder (S5 Fig). After making a pilot hole, the pedicle probe jig was changed to the screwdriver and PS insertion jig in the same manner described above.

### Postoperative evaluation

**Fig 1:**
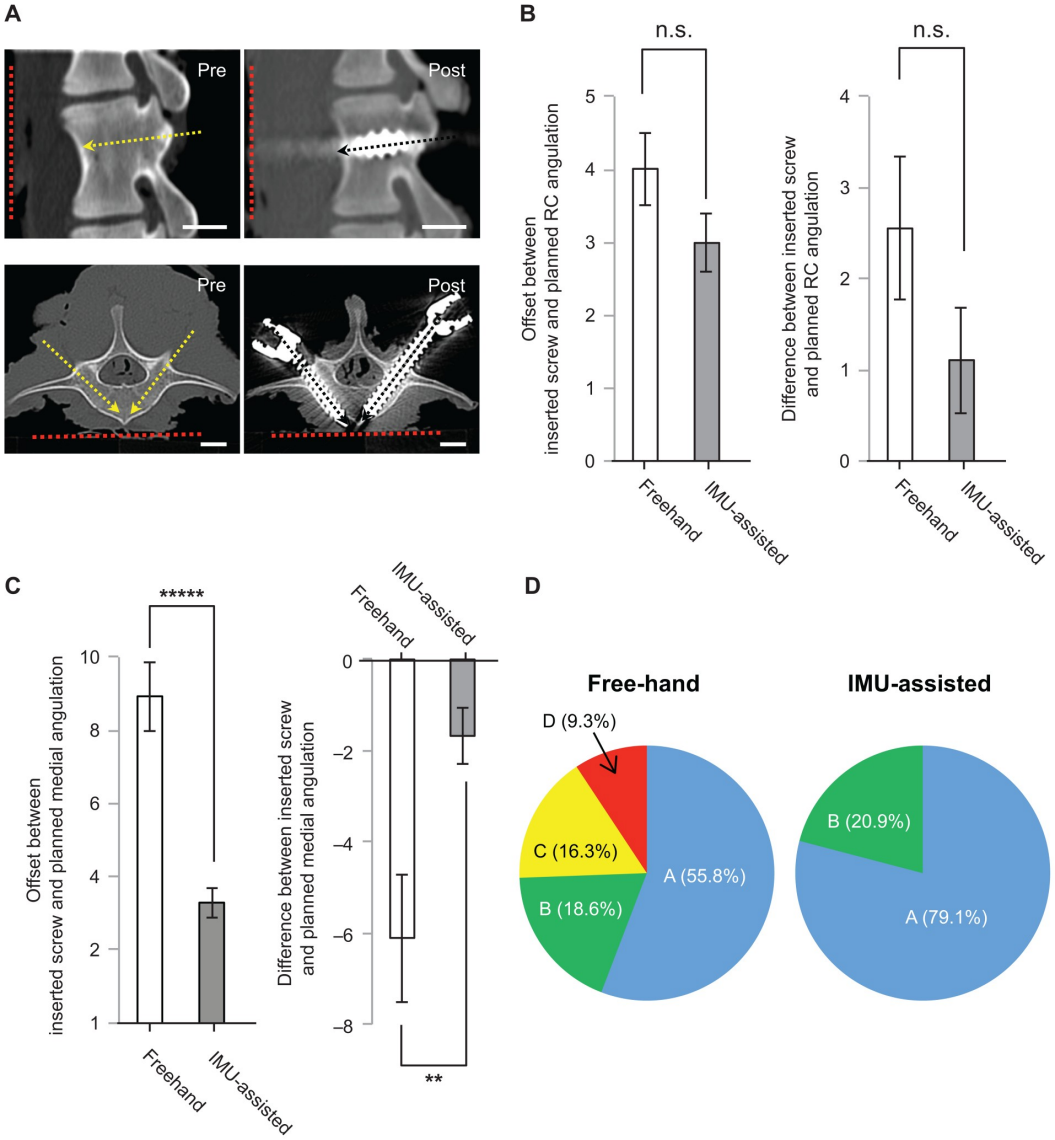
(a), Images of parasagittal (left) and arbitrary axial plane (right) obtained on pre- and postoperative CT scans. Scale bar: 10 um. Red dotted line indicates the surface of the board. Yellow dotted arrows indicate the planned trajectories of PSs. Black dotted arrows indicate the longitudinal axes of the inserted PSs. The left 2 panels show that the PS rostro-caudal angle was almost parallel to the planned angle. The right 2 panels show that the left PS medio-lateral angle deviated laterally relative to the planned angle, whereas the ML angle of the right PS was almost parallel to the planned angle. (b), Left), Mean offsets between the inserted PS and preoperatively planned rostro-caudal rotations about the transverse axis (see Material and methods) in the freehand and IMU-assisted groups. Right), Average difference between the inserted PS and preoperatively planned rostro-caudal rotation about the transverse axis (see Material and methods) in the freehand and IMU-assisted groups. n.s.: not significant. (c), Left),Mean offsets between the inserted PS and preoperatively planned medio-lateral rotations about the perpendicular axis (see Material and methods) in the freehand and IMU-assisted groups. Right), Average difference between the inserted PS and preoperatively planned medio-lateral rotations about the perpendicular axis (see Material and methods) in the freehand and IMU-assisted groups. *****: p < 0.00001, **: p < 0.01. (d), Pie charts demonstrating the proportion of positioning of PS in the freehand and IMU-assisted groups according to the Gertzbein and Robbins classification system.

A postoperative CT scan was performed, and rostro-caudal and medial angles of the implanted PSs were measured in MPR mode on a DICOM viewer. The angles were then compared to the preoperatively planned angles (Fig 1A). The screw trajectory was also checked for cortical bone breaches and evaluated according to the Gertzbein and Robbins classification system [27]. We evaluated the distance offset of the inserted screw EP and planned EP, 3-dimensional coordinates of the intersection point of the PS center axis and dorsal cortex of the base of the rostral articular process (i.e., EP-postop), TTP-postop, and rostral TRAP-postop were measured on a DICOM viewer to calculate the distances between the EP-postop and TRAP-postop.

### Sample sizes and statistical evaluation

The results are reported as means ± standard errors. When performing the one-tailed t-test with an alpha error of 5%, statistical power of 80%, and effect size from medium (0.5) to large (0.8) [28], 21 to 51 samples were necessary (R version 3.4.4 package pwr), so we aimed to obtain data from that number of samples.

**Fig 2:**
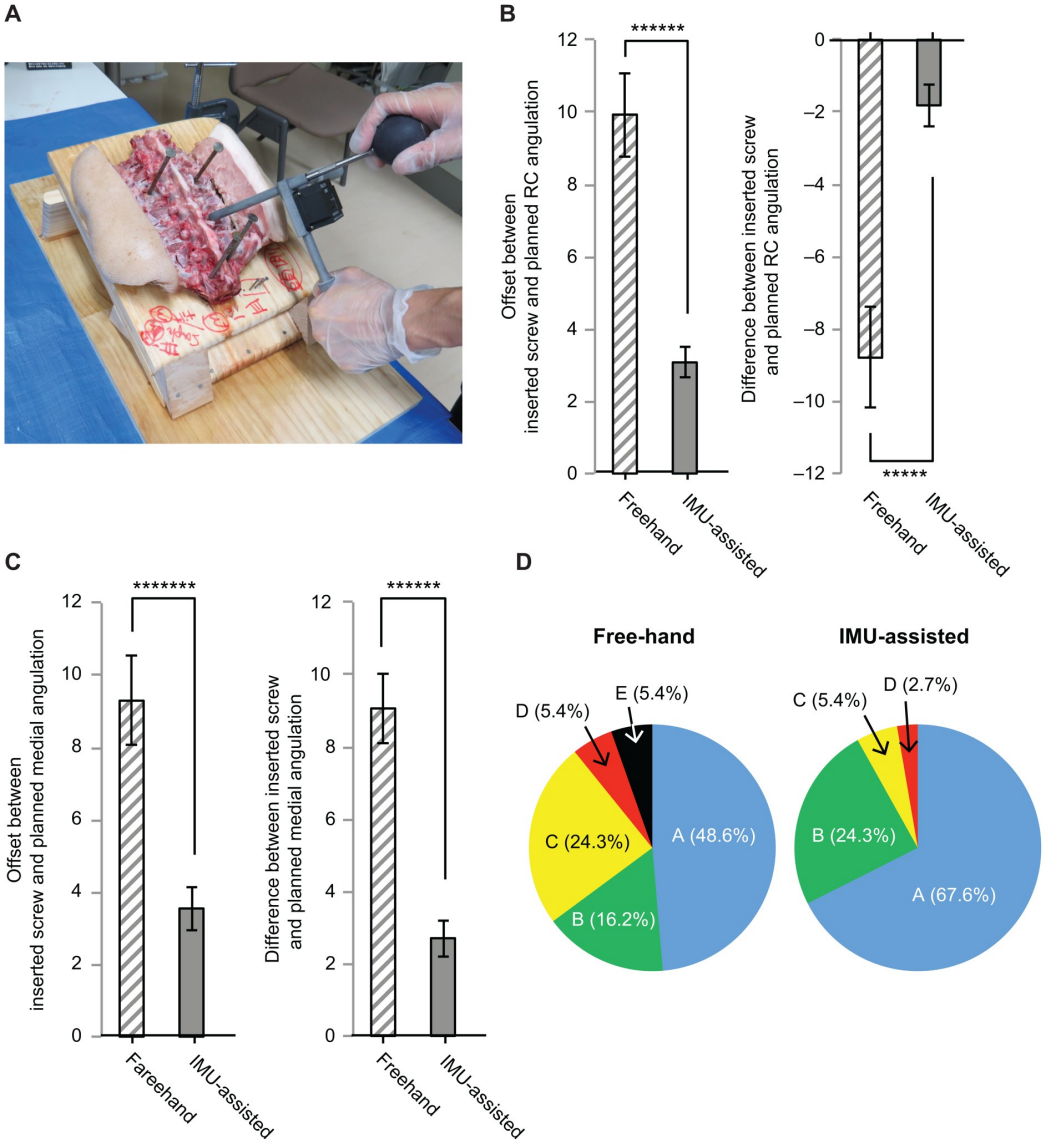
(a), Making a pilot hole for PS placement in the sample on a table tilted 20° about the transverse axis by using IMU assist. (b), Left), Mean offsets between the inserted PS and preoperatively planned rostro-caudal rotations about the transverse axis in the head-down-tilted samples. Right), Average difference between the preoperatively planned and inserted PS rostro-caudal rotations about the transverse axis (see Material and methods) in the head-down-tilted samples for the freehand and IMU-assisted groups. ******: p < 0.000001, *****: p < 0.00001. (c), Left), Mean offsets between the preoperatively planned and inserted PS medio-lateral rotations about the perpendicular axis in the head-down-tilted samples for the freehand and IMU-assisted groups. Right), Average difference between the preoperatively planned and inserted PS medio-lateral rotations about the perpendicular axis (see Material and methods) in the head-down-tilted samples for the freehand and IMU-assisted groups. *******: p < 0.0000001, ******: p < 0.000001. (d), Pie charts demonstrating the proportion of positioning of PS in the head-down-tilted samples in the freehand and IMU-assisted groups classified by the Gertzbein and Robbins classification system.

Screw placement was not performed when nails for fixing samples to a board were observed on the ideal trajectory at preoperative planning. The data were also excluded when the drilled EPs were found to be >2 mm away from the planned EPs in postoperative analyses of EP coordinates. Consequently, 86 screws were implanted into nine lumbar spine samples (L1–6) without rostral tilting (Fig 1), 43 with freehand technique and 43 with IMU-assisted technique, and a total of 74 screws were implanted into eight samples with rostral tilting (Fig 2), 37 with freehand technique, and 37 with IMU-assisted technique.

The freehand and IMU-assisted group results were compared with the independent 2-sample t-test (Figs 1B, 1C, 2B and 2C) and chi-squared test for categorical data (Figs 1D and 2D) by using Origin 8.6 (OriginLab corporation, MA, USA). The significance level was set to p = 0.05.

## Results

### Human angle estimation ability varied with the displayed angle

A previous study on estimating the theoretical accuracy requirement for PS placement demonstrated that if there was no translational error for the PS placement EP, the maximum permissible rotational error tolerance for avoiding pedicle wall perforation was <1° at several spines in a spinal pedicle geometric model [29]. On the basis of this result, we validated the angular offset between the angle randomly shown on the PC monitor with a unit angle of 1° and the angle manually reproduced by a digital protractor used by the observers (S1 Fig). The results demonstrated that the angular offset was relatively small when the displayed angle was near 0° or 90°. However, the angular offset increased as the displayed angle value increased from 0°, peaked when the displayed angle value was near 50°, and then gradually decreased as the displayed angle increased >60° (S1 Fig).

### Improved reproducibility of the preoperatively planned medial rotation about the perpendicular PS axis (better reproducibility of the planned PS trajectory) by IMU assist

The results of inaccurate angle estimation ability described above suggested that our just noticeable angle difference was not small enough for producing accurate medial rotation of the pedicle probe or PS driver at all vertebral levels, and the inaccuracy increased as the ideal medial rotational angle for PS placement into human vertebrae increased from 0° to 30°, as demonstrated in a previous paper [30].

Therefore, we hypothesized that the offset between the preoperatively planned and postoperatively measured implanted PS trajectories could be eliminated if the trajectory of the pedicle probe or screwdriver for PS placement was continuously monitored by using the IMU. No significant differences in the averaged planned rostro-caudal and medial rotation angles (Table 1), planned distance entry point-related parameters (Table 2), averaged PS diameters, narrowest pedicle width, and PS size-to-narrowest pedicle width ratio (Table 3) were observed between the freehand and IMU-assisted groups.

**Table 1:** Average planned rotational angles of the freehand and IMU-assisted groups with or without rostral tilt of the samples.

Planned rotation	Freehand (w/o tilt) n = 43	IMU (w/o tilt) n = 43	*p* value	Freehand (w/ tilt) n = 37	IMU (w/ tilt) n = 37	*p* value
**rostro-caudal (°)**	−3.81 ± 0.84	−3.79 ± 0.84	0.27	−28.2 ± 1.01	−28.9 ± 0.85	0.13
**medial (°)**	36.6 ± 0.94	34.6 ± 0.88	0.13	39.9 ± 0.93	37.8 ± 1.07	0.14

IMU, inertial measurement unit.

**Table 2:** Average differences between the postoperatively measured and planned distances between EP and TRAP or TTP.

Position displacement	Freehand (w/o tilt) n = 43	IMU (w/o tilt) n = 43	*p* value	Freehand (w/ tilt) n = 37	IMU (w/ tilt) n = 37	*p* value
**EP-TRAP (mm)**	1.25 ± 0.14	1.31 ± 0.15	0.79	1.13 ± 0.11	0.99 ± 0.85	0.44
**EP-TTP (mm)**	1.55 ± 0.14	1.29 ± 0.14	0.18	1.59 ± 0.23	1.27 ± 0.16	0.14

**Table 3:** Average diameters of the PS, narrowest width of the pedicle, and PS size-to-narrowest-pedicle width ratio.

Sizes and ratio	Freehand (w/o tilt) n = 43	IMU (w/o tilt) n = 43	*p* value	Freehand (w/ tilt) n = 37	IMU (w/ tilt) n = 37	*p* value
**PS diameter (mm)**	5.59 ± 0.12	5.69 ± 0.11	0.57	6.05 ± 0.13	5.98 ± 0.13	0.70
**pedicle width (mm)**	6.79 ± 0.11	6.87 ± 0.11	0.60	7.22 ± 0.13	7.21 ± 0.13	0.94
Ratio **(%)**	82.1 ± 0.48	82.5 ± 0.43	0.49	83.6 ± 0.45	82.8 ± 0.56	0.24

For all implanted PSs, the mean offsets between the preoperatively planned PS placement and postoperatively measured implanted PS rostro-caudal rotations about the transverse axis were 4.00° ± 0.50° in the freehand group vs. 2.99° ± 0.39° in the IMU-assisted group (mean ± standard error) (p = 0.0811) (Fig 1B, left). This result indicates that the IMU did not help reduce the offset between the preoperatively planned and implanted PS rotations. The average difference between the preoperatively planned and implanted PS rostro-caudal rotations were 2.29° ± 0.74° in the freehand group vs. 1.11° ± 0.58° in the IMU-assisted group (p = 0.145) (Fig 1B, right). This result indicates that compared with the preoperatively planned rostro-caudal rotation, the PS tended to rotate rostrally with freehand technique, and use of the IMU could not significantly reduce the PS rostral rotational deviation.

On the other hand, the mean offsets between the preoperatively planned and postoperatively measured medial rotations about the perpendicular PS axis were 8.93° ± 0.95° in the freehand group vs. 3.29 ± 0.42° in the IMU assist group, which indicated that the planned PS medial rotation was reproduced significantly better by the IMU-assisted technique than by the freehand technique (p < 0.00001) (Fig 1C, left). The arithmetic mean difference between the preoperatively planned and postoperatively measured implanted screw medial rotations were −6.11° ± 1.41° in the freehand group vs. −1.68° ± 0.61° in the IMU-assisted group (p = <0.01) (Fig 1C, right). This result indicates that the implanted PS tended to tilt laterally with the freehand technique relative to the preoperatively planned PS angle, and the IMU significantly reduced the PS lateral rotational deviation.

### IMU assist significantly increased the rate of acceptable Gertzbein and Robbins grades A and B for pedicle cortical layer breaches

Breaches of the cortical layer of pedicles in the freehand and IMU-assisted groups were evaluated according to the Gertzbein and Robbins criteria [27] (Fig 1D). In the freehand group, rates of grade A (an intrapedicular screw without breach of the cortical layer of the pedicle) and grade B (a screw that breaches the pedicle cortical layer by ≤2 mm) were 55.8% (24/43) and 18.6% (8/43), respectively. On the other hand, the rates of grade A and B in the IMU-assisted group were 79.1% (34/43) and 20.9% (9/43), respectively. No case of grade C, D, or E was observed in the IMU-group; however, grade C (penetration < 4 mm) and grade D (penetration < 6 mm) were observed in 16.3% (7/43) and 9.3% (4/43) in the freehand group, respectively. The trajectories of grades C and D in the freehand group had a high probability of lateral direction positioning (91%, 10/11). No PS in both groups penetrated the rostral or caudal pedicle walls (Table 4). Compared with the freehand technique, IMU assist significantly increased the rate of acceptable grades A and B (chi-square value: 12.6, p < 0.001).

**Table 4:** Screw positioning and direction of unacceptable wall penetration in the freehand and IMU-assisted groups according to the Gertzbein and Robbins classification system.

		Freehand (without tilt) n = 43	IMU (without tilt) n = 43	Freehand (with tilt) n = 37	IMU (with tilt) n = 37
*acceptable positioning		32	43	24	34
**unacceptable positioning		11	0	13	3
**Wall penetration direction**	**Medial**	1	0	3	0
	**Lateral**	10	0	10	3
	**Rostral**	0	0	0	0
	**Caudal**	0	0	0	0

*Acceptable positioning corresponds to grades A and B based on the Gertzbein and Robbins classification system.

**unacceptable positioning corresponds to grades C, D, and E based on the Gertzbein and Robbins classification system.

### Does further pedicle tilting decrease screw placement accuracy? If so, can IMU assist prevent this decreased accuracy? Improved accuracy was observed under specific conditions

In the range of 0° to 60°, angular offset between the displayed angle and manually reproduced angle increased when the displayed angle value increased from 0°, as shown in S1 Fig. From this result, it was expected that adding head-up or head-down tilt to the samples with caudal or rostral tilt to pedicles in the sagittal plane could result in increasing rostro-caudal angular offset in the sagittal plane between the planned tilt and freehand technique-implanted screw tilt. To test this hypothesis, screw insertions were performed on samples mounted on a table with 25° head-down tilt (Fig 2A). We also determined if IMU-support could still prevent the decrease in accuracy of rostro-caudal angular offset of inserted PSs.

Between the freehand and IMU-assisted groups, no significant differences in the averaged planned rostro-caudal and medial rotational angles were observed (Table 1), the planned EP-related distance parameters (Table 2), averaged PS diameters, narrowest pedicle width, and the PS size-to-narrowest pedicle width ratio (Table 3).

Among all implanted PSs, the mean offset between the preoperatively planned PS and postoperatively measured implanted PS transverse axis of the rostro-caudal rotations were 9.93° ± 1.16 (p < 0.000001) in the freehand group vs. 3.08° ± 0.42° (p <0.000001) in the IMU-assisted group (Fig 2B, left). In this case, IMU assist helped prevent increasing rostro-caudal offset between the planned and implanted PS rotations. The average difference between the preoperatively planned and implanted PS rostro-caudal rotations in the head-down-tilted samples were −8.80° ± 1.39° in the freehand group vs. −1.82° ± 0.59° in the IMU-assisted group (p < 0.00001) (Fig 2B, right). These results indicated that IMU assist significantly prevented the tendency toward dorsal tilting of PS observed in the freehand group revealed by head-down tilting of samples.

The mean offsets between medial rotations about the perpendicular axis of the preoperatively planned and postoperatively measured implanted PS in the head-down-tilted samples were 9.06° ± 0.76° in the freehand group vs. 2.92° ± 0.48° in the IMU-assisted group, which indicated that the IMU-assisted technique prevented deterioration of accuracy of the planned medial rotation of PS (p < 0.0000001) (Fig 2C, left). The arithmetic mean of the difference in the medial rotations about the perpendicular axis between the preoperatively planned and postoperatively measured implanted screw were 9.06° ± 0.96° in the freehand group vs. 2.71° ± 0.51° in the IMU-assisted group (p < 0.000001) (Fig 2C, right). This result indicates that the implanted PS tended to tilt medially with freehand technique relative to the angle of the preoperatively planned angle in the head-down tilted sample, and the IMU again reduced the medial rotational PS deviation.

### IMU assist decreased PS breach of pedicle cortical layers in head-down-tilted samples under certain conditions

The grade A and grade B (Gertzbein and Robbins classification) rates in the freehand group for head-down tilting were 48.6% (18/37) and 16.2% (6/37), respectively, whereas the grade A and B rates in the IMU-assisted group were 67.6% (25/37) and 24.3% (9/37), respectively (Fig 2D). Grade E (5.4%, 2/37) (pedicle wall penetration ≥ 6 mm) that was not observed in the freehand group without adding head-down tilting of the spine was revealed with head-down tilting in addition to grade C (5.4%, 2/37) and D (5.4%, 2/37). Grade C (5.4%, 2/37) and grade D (2.7%, 1/37) that were not observed in the IMU-assisted group without adding sagittal tilting were observed with head-down tilting. In the freehand group, head-down tilting significantly increased the unacceptable rate relative to the rate without head-down tilting (chi-square: 4.86, p < 0.05), whereas there were no significant differences in the acceptance rates of the IMU-assisted group with and without head-down tilting (chi-square: 3.62, p = 0.057). Even with head-down tilting, IMU assist significantly increased the acceptable rate (chi-square: 7.97, p < 0.01) relative to that in the freehand group. After adding head-down tilt, critical medial wall penetrations potentially associated with irreversible neurological complications were observed in some cases in the freehand group (8.1%, 3/37) that were not observed with IMU assist. No screws penetrated the rostral or caudal pedicle walls in either the freehand or IMU-assisted groups (Table 4).

## Discussion

Three elements are important for successful PS placement: 1) tactile feedback from cancellous bone surrounding pedicle cortical bone, 2) accuracy of EP coordinates, and 3) accuracy of pedicle probe and screwdriver trajectories to the vertebral body. However the tactile sensation from cancellous bone for an operator is hard to describe verbally and cannot be double-checked by assistants. Moreover, tactile feedback from cancellous bone is difficult to obtain when the target pedicle or portion adjacent to the pedicle exhibits sclerotic change. Therefore, it is important, especially for an inexperienced spine surgeon, to pay careful attention to the coordinates of EP and trajectories for probing or screwdriving. Regarding the EP in the traditional procedure for PS placement, several clear anatomical landmarks (e.g., transverse process, accessory process, superior articular process) can help define the EP and can be double-checked by the operator and assistants.

On the other hand, regarding the trajectories of pedicle probing and screw driving, these parameters cannot be measured easily and accurately; therefore, operators using conventional freehand technique must adjust these parameters by intuition to some extent. When trying to reproduce a visually displayed planned angle without any tool, the offset between the angle to be reproduced and reproduced angle in practice must be noticed initially. In other words, it is expected that the smaller the just noticeable difference (JND) against the angle to be reproduced, the better the reproducibility of that angle. About JND, a previous paper demonstrated that JND in an angle increased with an increasing angle from 0° to 45° and then diminished as the angle size increased from 45° to 90°, potentially depending on orthogonal internal reference frame (vertical and horizontal lines) acquired by postnatal visual experience and Weber’s law, as applied to perception of an angle change [31]. Our angle reproducibility results are comparatively consistent with the results described above.

In PS placement planning in humans, the goal is to match the PS trajectory with the pedicle longitudinal axis. Consequently, the ideal trajectory is on the transverse plane arbitrarily rotated about the transverse axis. In the lumbar spine, for example, the ideal trajectory is generally on the transverse plane parallel to the upper endplate of the vertebral body. In that case, the surgeon initially rotates the pedicle probe about the transverse axis until the rostro-caudal rotation of the probe matches the preoperatively planned rotation and then rotates the probe knob laterally about the perpendicular axis until the pedicle probe longitudinal axis matches the pedicle longitudinal axis. Therefore, the probe and screwdriver rotations demonstrated poor reproducibility the 2 steps that can doubly potentiate the trajectory offset between the implanted PS and preoperative planning trajectories. Furthermore, because the surgeon usually looks down on the pedicle probe or screwdrivers, accurate evaluation of the rostro-caudal and medial rotation of pedicle probe is nearly impossible.

It is not so surprising that rostro-caudal and medial rotational errors could be reduced by using the IMU to enable trajectory monitoring of surgical tools. However, in this study, the average upper endplate caudal rotation about the transverse axis of our samples was <4° (Table 1), and the IMU did not increase the accuracy of the planned PS rostro-caudal rotational angles. This result could be reasonably explained by the result demonstrated in S1 Fig and the previous paper [31]; namely, average offsets between the displayed and reproduced angles with freehand technique were smaller when the displayed angles were smaller, whereas the average medial rotation of the pedicle longitudinal axis about the perpendicular axis was >30° (Table 1). As expected again from the results in S1 Fig, the average offsets between the freehand displayed and reproduced angles were significantly larger for displayed angles from 30°–50° than for those from 0°−30°, and the IMU significantly helped increase the accuracy of the planned PS medial rotation. Furthermore, when vertebral rotations about the transverse axis were intentionally increased by 20°, and the reproducibility of the planned rostro-caudal rotation about the transverse axis was expected to decrease, as shown in S1B Fig, the situation mimicked high-grade spondylolisthesis and/or discal or vertebral wedging deformity, and use of the IMU again improved the accuracy of planned rostro-caudal rotation of PSs observed in the freehand groups (Fig 2B). In this manner, depending on the range of planned rotation, there were situations in which IMU assist was very useful, such as with a larger planned trajectory, and situations in which the IMU assist did not function very effectively, such as with a smaller planned trajectory. These findings are important to consider when deciding if the IMU will be advantageous.

Previous studies have demonstrated that the acceptance rates of PS placement in the thoracic and/or lumbar spine ranging from 62% to 91% without using navigation systems [32–37]. Although a simple comparison is impossible because our samples were not the same as those in previous studies, the clinically acceptable rates in the freehand groups were 74% without and 65% with head-down tilting of samples, which are within the range reported in the previous studies, whereas the acceptance rates in the IMU-assisted group were 100% without and 92% with head-down tilting, which were superior to those previously reported, suggesting that IMU assist could increase PS placement safety.

There were several limitations in our study. First, the experimental results were obtained from porcine lumbar spines. Although the pig is a well-accepted model for research involving PSs [38–41], the morphological features of the spine do not completely match those of the human lumbar spine [42]. Therefore, the accuracy and reproducibility advantages of using an IMU should be confirmed in human cadaver spines. Second, the IMU was fixed on the jig without sterilization in this study. In actual use, the IMU must be sterile, but it can be stored in a container attached to the jig for repeated sterilization. Thus, the material and form of the jig should be considered before applying this method in practice. Although the exact cost of the usable jig is yet unclear, its cost should be less than 1000 USD, which is much less expensive than that of imaging navigation or robot-assisted systems. Third, the PS diameter in this study relative to the narrowest width of the pedicle was relatively large (PS diameter/pedicle width > 80%). Practically, however, the PS diameter-to-pedicle width ratio is not usually so large and sometimes <50% in human spine surgery, especially in lower lumbar spines. Thus, it is possible that the pedicle wall penetration ratio demonstrated in our study is exaggerated in comparison with the ratios observed in previous studies. Fourth, micro-rotations of the spine caused by applying loads while pedicle probing or PS insertion caused difficulty in measuring rotational angles of the pedicle probe or PS driver relative to the vertebral longitudinal and transverse axes even with the IMU assist. To avoid these rotations of the spine, surgeons using IMU should measure the rotation when these loads have been released. Fifth, we performed the PS placement experiments in porcine lumbar spine pedicles with relatively large diameters. Thus, it will be useful in the future to test whether the same advantage provided by IMU assistance is observed in narrower pedicles corresponding to the situation in which the PS is placed in the human cervical or mid-thoracic spine. In the future, it would also be interesting to compare IMU-assisted and CT-based navigation system with respect to the accuracy, cost-effectiveness, and operation time of PS placement. As a relatively low-cost device for avoiding wall penetration of PSs in addition to IMU-mounted jigs, the electrical conductivity measurement device for monitoring changes in impedance at the tip of the probe to detect iatrogenic pedicle perforation is commercially available, and such a device was developed based on a different concept from ours. These devices can be combined and simultaneously used, synergistically increasing the safety of PS placement. Although there are several issues to be solved before the IMU-assist technique can be clinically introduced for PS placement in human spine surgery, this technique may bring benefits to less developed countries or institutions that do not have access to high-cost navigation systems for PS placement.

## Conclusions

This study demonstrated the inaccuracy of human angle estimation ability in freehand PS placement technique. Monitoring of the pedicle probe and PS driver 3-dimensional trajectories by using the IMU-assisted technique improved the accuracy and safety of PS placement, especially when the rostro-caudal and/or medial rotation angle to be reproduced is large.

## Supporting information

S1 Fig Evaluation of angle reproducibility. (upper), Schematic diagram of evaluation of angle reproducibility in the range of 0° to 90° randomly demonstrated on a PC monitor. Inset demonstrates a screenshot of PC monitor displaying an angle out of 91 randomly demonstrated angle. (lower), Plot of mean offsets between measures of angles on the digital protractor reproduced by observers and on the PC monitor versus true measures of angles displayed on the PC monitor. Curve superimposed on the plot corresponds to the approximated curve obtained by least squares fitting (y = −0.0014x^2^ + 0.1495x + 0.0759, R^2^ = 0.5991, vertex of the approximating curve = 53.4°). The polynomial degree was set to 2 on the basis of the idea that 0° and 90° were the internal reference frame (also see the results).
(TIFF)

S2 Fig IMU-mounted jig for pedicle probe. Two handles are attached to the IMU platform, and the attitudes of these 2 handles correspond to the sagittal and transverse axes (also see the Method section). Inset illustration demonstrates that the axes of the 2 handles correspond to caudal-rostral and medial-lateral rotation axes when 1 handle and the others were set parallel and perpendicular to the spinal body axis and the outer cylinder was set perpendicular to the ground.
(TIF)

S3 Fig Medio-lateral and rostro-caudal inclination. Sagittal axis of the IMU corresponds to the longitudinal axis of the spinous process tip. Note that the medical inclination angle on the plane rotated around the transverse axis (β on S3 Fig; e.g., the plane parallel to the spinal upper endplate) does not correspond to the angle of the attitude of the outer cylinder projected onto the transverse plane (γ in S3 Fig). The inclination angles measured by the IMU correspond to α and γ.
(TIF)

S4 Fig Entry point for PS placement (dotted circle). See also the details in the Method and material section. (i) Supraspinous ligament, (ii) L3 caudal articular process, (iii) cranial articular process, (iv) transverse process, (*) tip of the L4 cranial articular process, (**) tip of the transverse process. Partial resection of the cranial articular process was needed (filled arrow head) to avoid skiving [43] in which the pedicle probe or screw tends to deviate caudally and laterally from the planned entry point.
(TIF)

S5 Fig Making a pilot hole for PS placement by using IMU assist. See also the Method and material sections. The bottom surface of the sample is horizontally cut to obtain a volume of soft tissues, and the sample is tightly fixed with >4 nails to a 3-cm thick wood board with a diameter of 5 mm to avoid tilting or rotating of the sample against the board.
(TIF)

S1 File Evaluation method for the angle estimation ability. (DOCX)
