# Supplementary figures and images for: Use of an inertial measurement unit sensor in pedicle screw placement improves trajectory accuracy
Source: PLoS One. 2020 Nov 16;15(11):e0242512. doi: 10.1371/journal.pone.0242512 (PMC7668595; doi:10.1371/journal.pone.0242512)

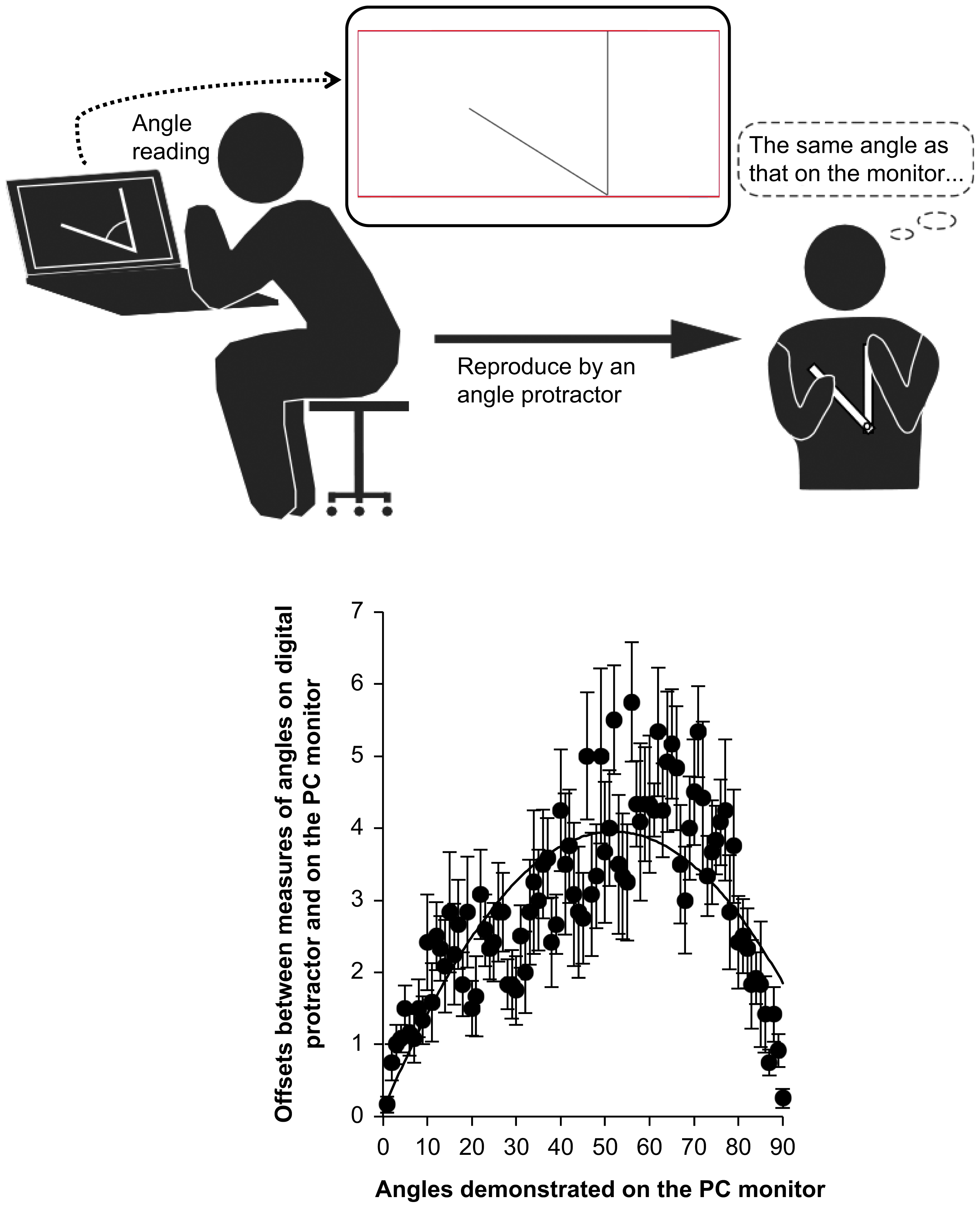

Supplement: S1 Fig — (upper), Schematic diagram of evaluation of angle reproducibility in the range of 0° to 90° randomly demonstrated on a PC monitor. Inset demonstrates a screenshot of PC monitor displaying an angle out of 91 randomly demonstrated angle. (lower), Plot of mean offsets between measures of angles on the digital protractor reproduced by observers and on the PC monitor versus true measures of angles displayed on the PC monitor. Curve superimposed on the plot corresponds to the approximated curve obtained by least squares fitting (y = −0.0014x2 + 0.1495x + 0.0759, R2 = 0.5991, vertex of the approximating curve = 53.4°). The polynomial degree was set to 2 on the basis of the idea that 0° and 90° were the internal reference frame (also see the results). (TIFF) [file pone.0242512.s001.tiff]

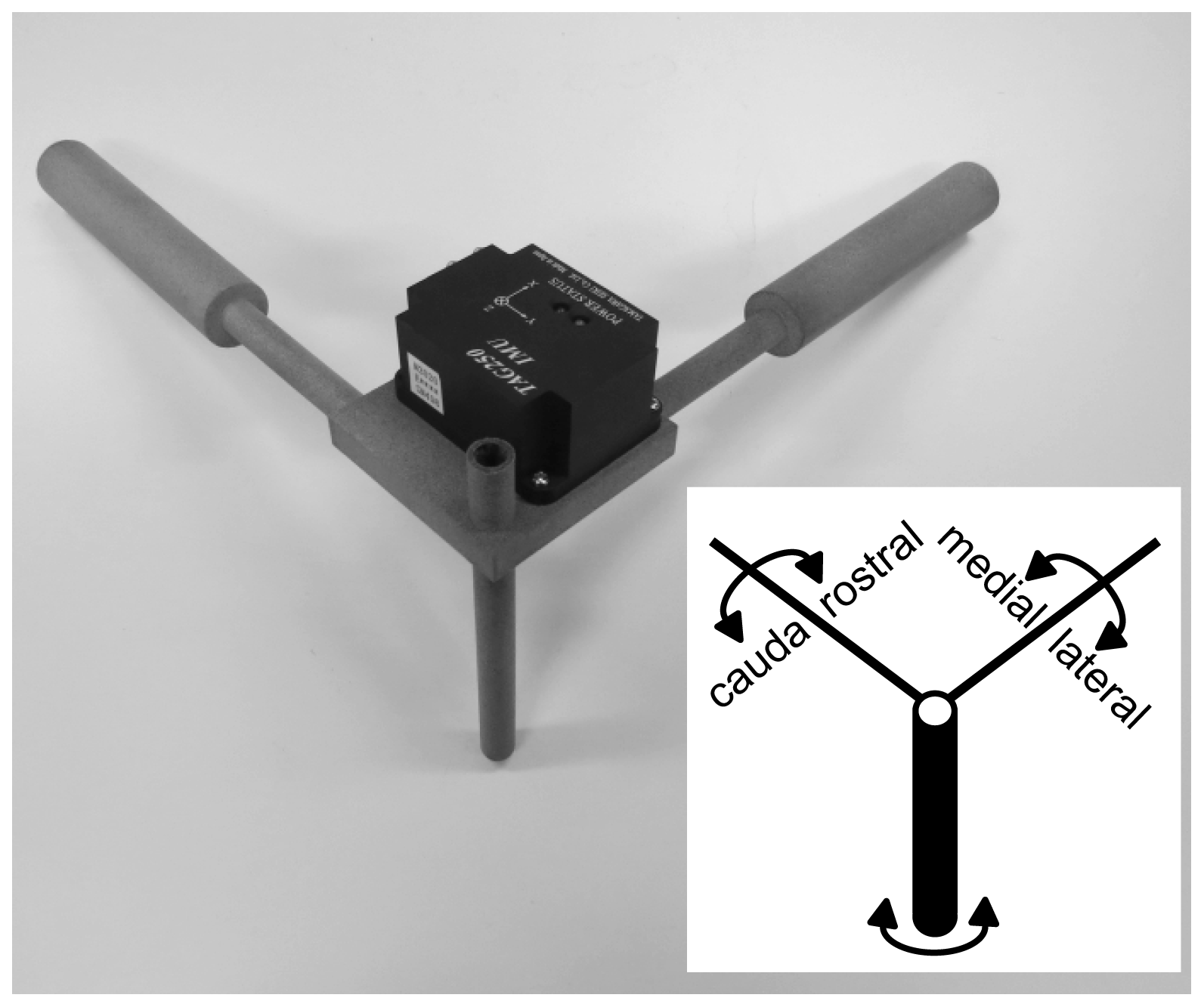

Supplement: S2 Fig — Two handles are attached to the IMU platform, and the attitudes of these 2 handles correspond to the sagittal and transverse axes (also see the Method section). Inset illustration demonstrates that the axes of the 2 handles correspond to caudal-rostral and medial-lateral rotation axes when 1 handle and the others were set parallel and perpendicular to the spinal body axis and the outer cylinder was set perpendicular to the ground. (TIF) [file pone.0242512.s002.tif]

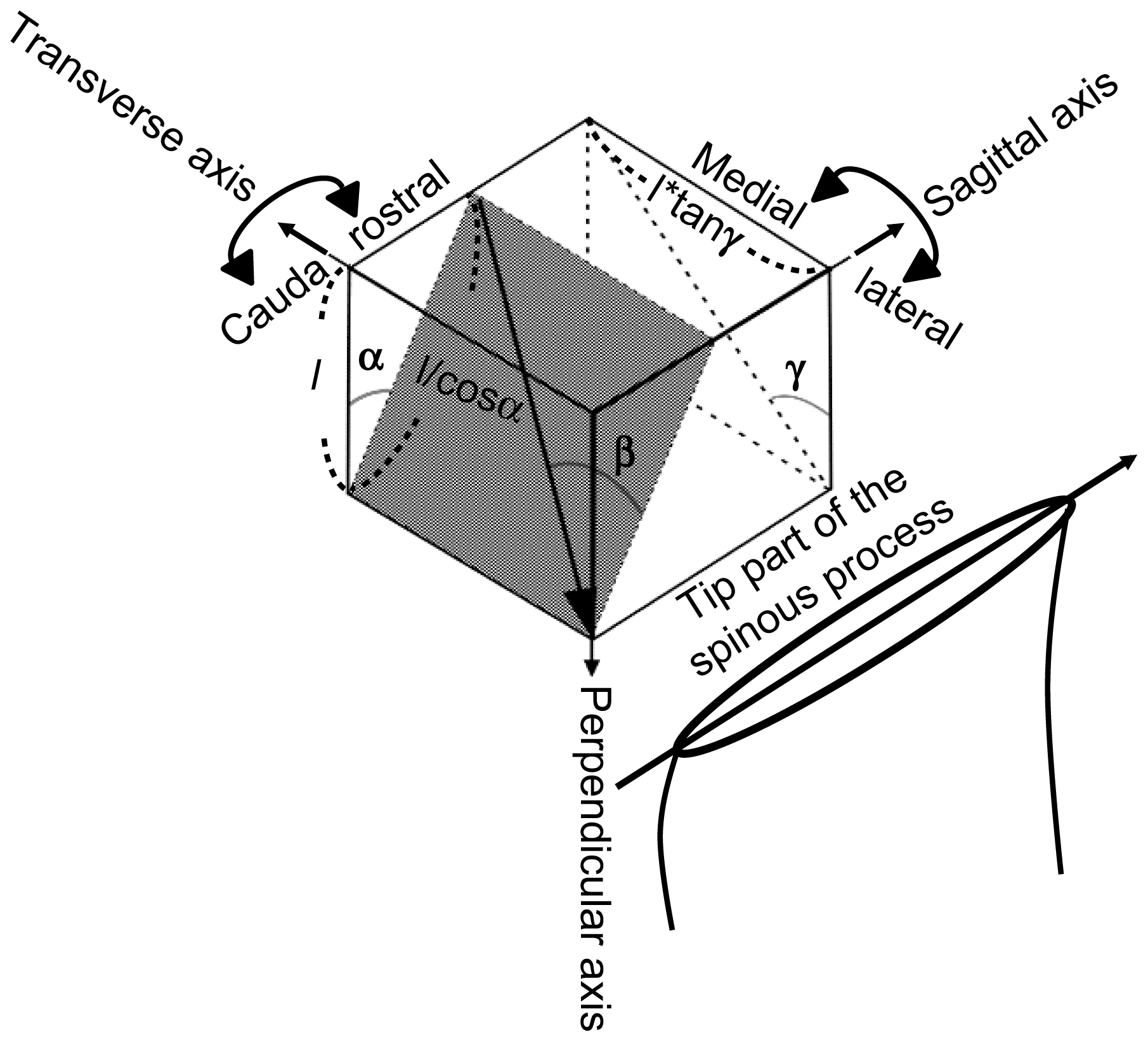

Supplement: S3 Fig — Sagittal axis of the IMU corresponds to the longitudinal axis of the spinous process tip. Note that the medical inclination angle on the plane rotated around the transverse axis (β on S3 Fig; e.g., the plane parallel to the spinal upper endplate) does not correspond to the angle of the attitude of the outer cylinder projected onto the transverse plane (γ in S3 Fig). The inclination angles measured by the IMU correspond to α and γ. (TIF) [file pone.0242512.s003.tif]

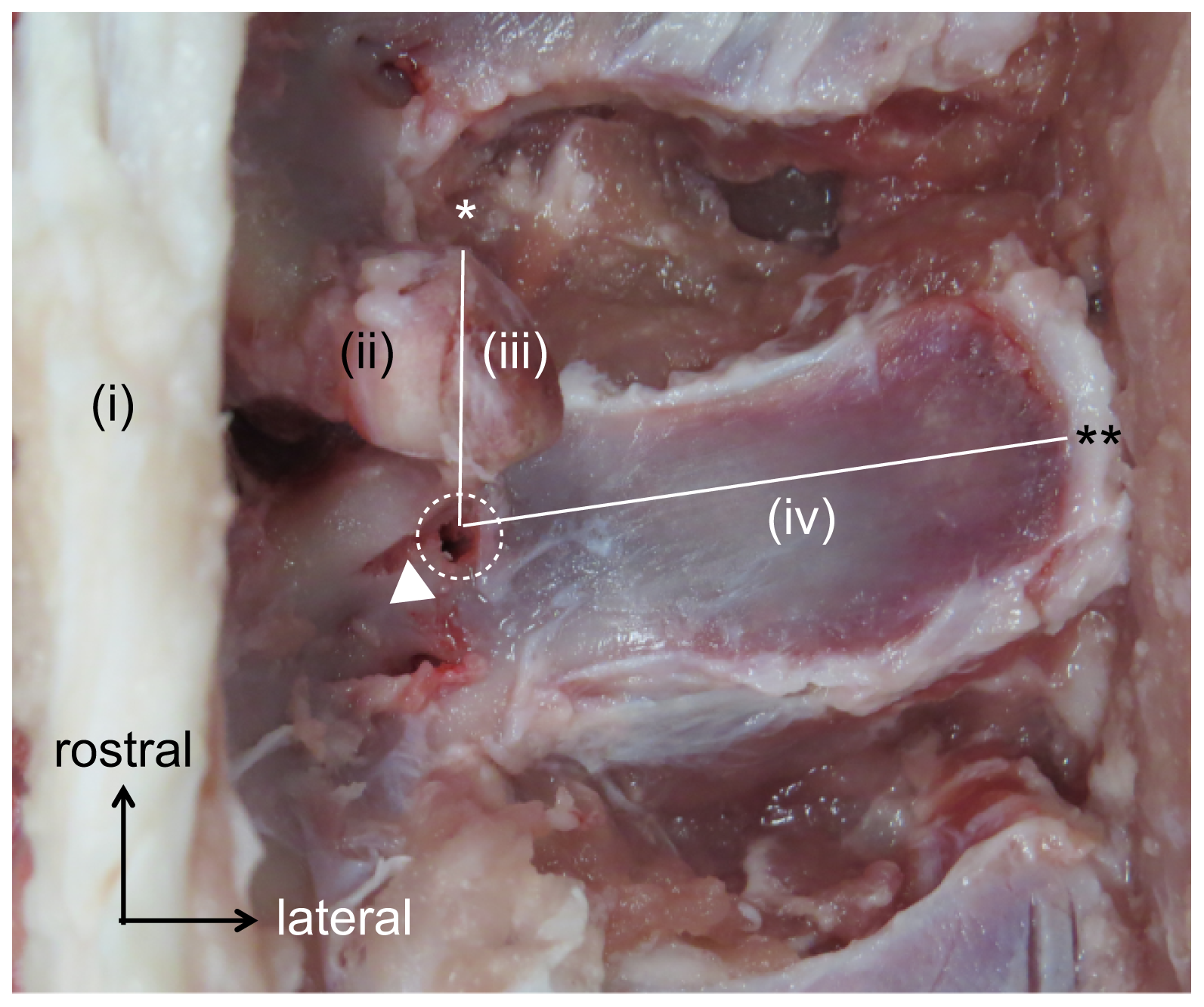

Supplement: S4 Fig — See also the details in the Method and material section. (i) Supraspinous ligament, (ii) L3 caudal articular process, (iii) cranial articular process, (iv) transverse process, (*) tip of the L4 cranial articular process, (**) tip of the transverse process. Partial resection of the cranial articular process was needed (filled arrow head) to avoid skiving [43] in which the pedicle probe or screw tends to deviate caudally and laterally from the planned entry point. (TIF) [file pone.0242512.s004.tif]

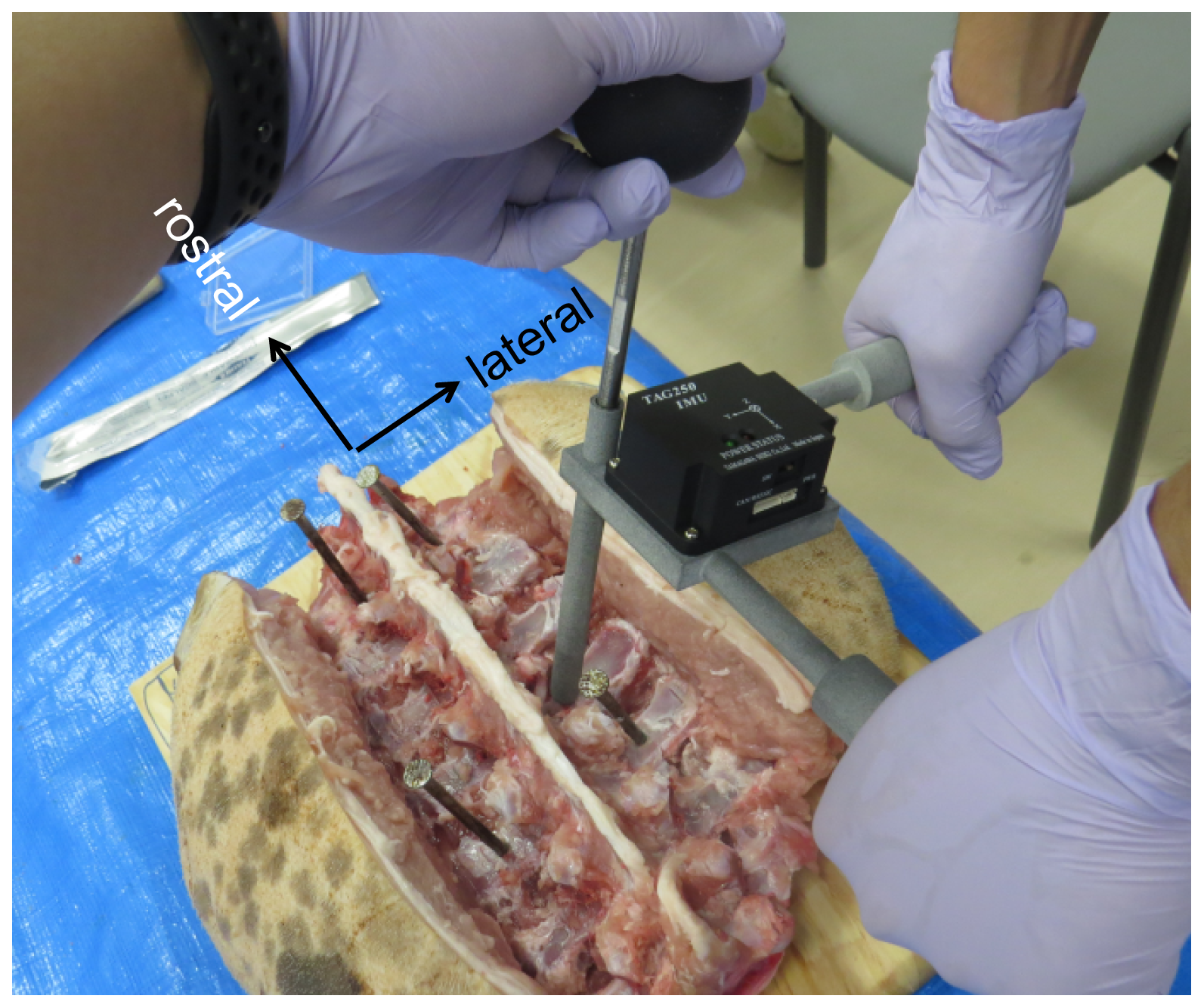

Supplement: S5 Fig — See also the Method and material sections. The bottom surface of the sample is horizontally cut to obtain a volume of soft tissues, and the sample is tightly fixed with >4 nails to a 3-cm thick wood board with a diameter of 5 mm to avoid tilting or rotating of the sample against the board. (TIF) [file pone.0242512.s005.tif]
