# Supplementary material for: Use of an inertial measurement unit sensor in pedicle screw placement improves trajectory accuracy
Source: PLoS One. 2020 Nov 16;15(11):e0242512. doi: 10.1371/journal.pone.0242512 (PMC7668595; doi:10.1371/journal.pone.0242512)
Supplement: S1 File — (DOCX) [file pone.0242512.s006.docx]

**S1 File. Evaluation method for the angle estimation ability**

Although a 3-dimensional numerical direction value of a pedicle probe or screw in PS insertion cannot be known in conventional freehand procedure, for accurate PS placement, it is sufficient if the manually reproduced attitude angle of a pedicle probe or screw relative to the vertebral bodies is the same as that of the preoperatively planned 3-dimensional directions. Therefore, we evaluated how accurately the observers could reproduce the angle on the PC monitor described below by using a digital protractor consisting of two 20-cm rulers (Zhejiang Beilong Tool Co. Ltd.). Then, the offsets between the angles reproduced by the observers and the true angles on the PC monitor were analyzed.

Angles in the range of 0° to 90° bounded by 2 lines of equal length (15 cm) were displayed on a computer monitor: one was vertical and always presented in the same position, and the other was connected at the bottom of the vertical line and tilted counterclockwise toward the center. This angle configuration was presented on the PC monitor randomly with a unit angle of 1°. Because previous studies have demonstrated that discrimination thresholds for angles bounded by 2 lines of different length were ≤3 times lower than those for those bounded by 2 lines of equal length [1] and that an angle embedded in a scalene triangle is judged to be substantially smaller than the same angle embedded in an isosceles triangle [2], the lines defining the displayed angles were designed to be the same length in our study.

The distance between the observer’s face and monitor was 40 to 80 cm. If the line of sight of an observer was not perpendicular to the plane (PC monitor) on which the angles were presented and if an observer observed angles from diagonally above or below, angle estimation could have been more difficult; thus, the PC monitor was set so that the line of sight of the observer was as much perpendicular to the PC monitor as possible.

Two lines bounding the angle were produced randomly by using custom-written software on a 13.3-inch monitor of a laptop computer (NEC, Japan). The spatial resolution was 2560 × 1440 pixels.

[1] Kennedy GJ, Orbach HS, Loffler G. Effects of global shape on angle discrimination. Vision Res 2006;46: 1530-9. [doi: 10.1016/j.visres.2005.06.003](https://doi.org/10.1016/j.visres.2005.06.003).

[2] Kennedy GJ, Orbach HS, Loffler G. Global shape versus local feature: an angle illusion. Vision Res 2008;48: 1281-9. [doi: 10.1016/j.visres.2008.03.003](https://doi.org/10.1016/j.visres.2008.03.003).
